# Supplementary material for: Aberrant expression of two miRNAs promotes proliferation, hepatitis B virus amplification, migration and invasion of hepatocellular carcinoma cells: evidence from bioinformatic analysis and experimental validation
Source: PeerJ. 2020 Apr 29;8:e9100. doi: 10.7717/peerj.9100 (PMC7195830; doi:10.7717/peerj.9100)
Supplement: Supplemental Information 1 [file peerj-08-9100-s001.docx]

Table S1 Primers sequences for qRT-PCR

| Genes | | Sequences |
| --- | --- | --- |
| miR-221-3p | loop primer | 5'-GTCGTATCCAGTGCAGGGTCCGAGGTATTCGCACTGGATACGACGCACCCCT-3’ |
|  | forward | 5'-TGCGCGCAGGGACAGCAAGCA-3’ |
|  | reverse | 5'-CCAGTGCAGGGTCCGAGGTATT-3' |
| miR-375 | loop primer | 5'-GTCGTATCCAGTGCAGGGTCCGAGGTATTCGCACTGGATACGACTCACGCGA-3' |
|  | forward | 5'-TGCGCTTTGTTCGTTCGGCTCA-3' |
|  | reverse | 5'-CCAGTGCAGGGTCCGAGGTATT-3' |
| U6 | forward | 5'-CGCTTCGGCAGCACATATAC-3’ |
|  | reverse | 5'-AAATATGGAACGCTTCACGA-3’ |
