# Supplementary figures and images for: Aberrant expression of two miRNAs promotes proliferation, hepatitis B virus amplification, migration and invasion of hepatocellular carcinoma cells: evidence from bioinformatic analysis and experimental validation
Source: PeerJ. 2020 Apr 29;8:e9100. doi: 10.7717/peerj.9100 (PMC7195830; doi:10.7717/peerj.9100)

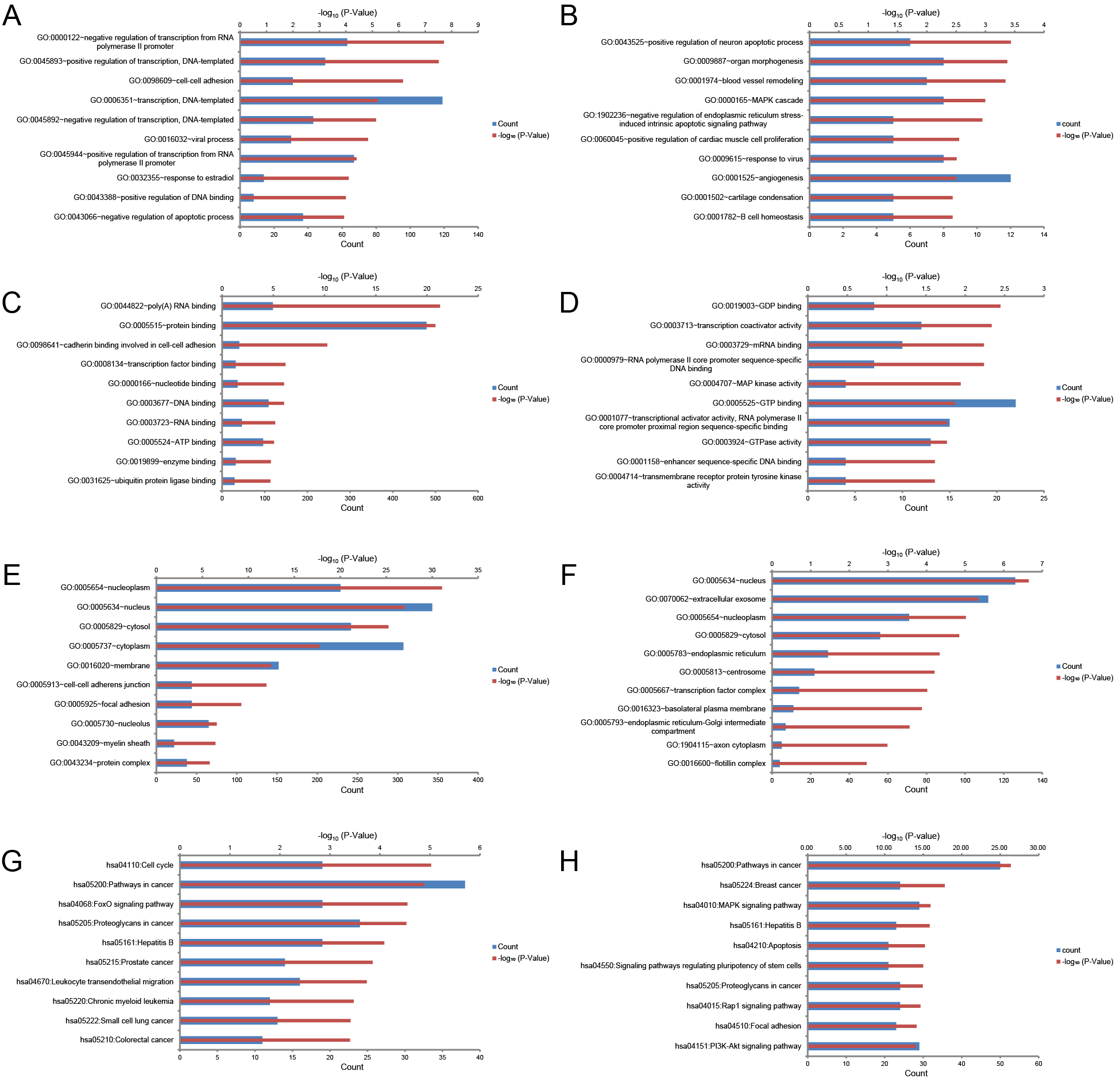

Supplement: Supplemental Information 2 — The top ten enriched (A) biological process, (C) molecular function, (E) cellular component, and (G) KEGG pathways analysis of target gene sets predicted by three upregulated miRNAs ranked by –log10(P-value); the top ten enriched (B) biological process, (D) molecular function, (F) cellular component, and (H) KEGG pathways analysis of target gene sets predicted by three downregulated miRNAs ranked by –log10(P-value). Red represented the –log10(P-value) of each cluster, blue represented enriched gene number of each cluster. [file peerj-08-9100-s002.png]

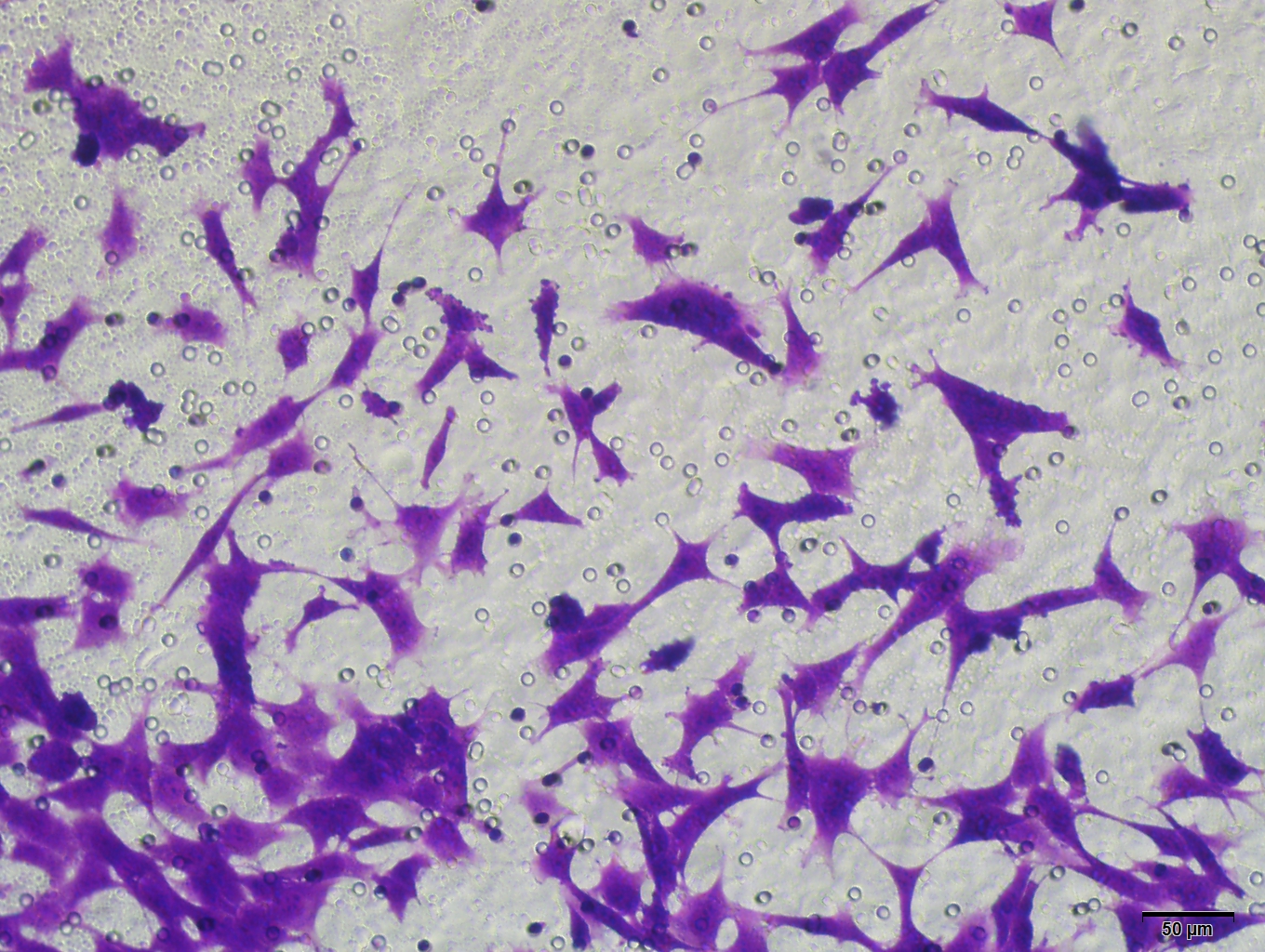

Supplement: Supplemental Information 3 — Figure data and images of WB, wound heal assay, and transwell assay [file peerj-08-9100-s003.zip › Raw data/Tanswell/Fig.7C--NC.jpg]

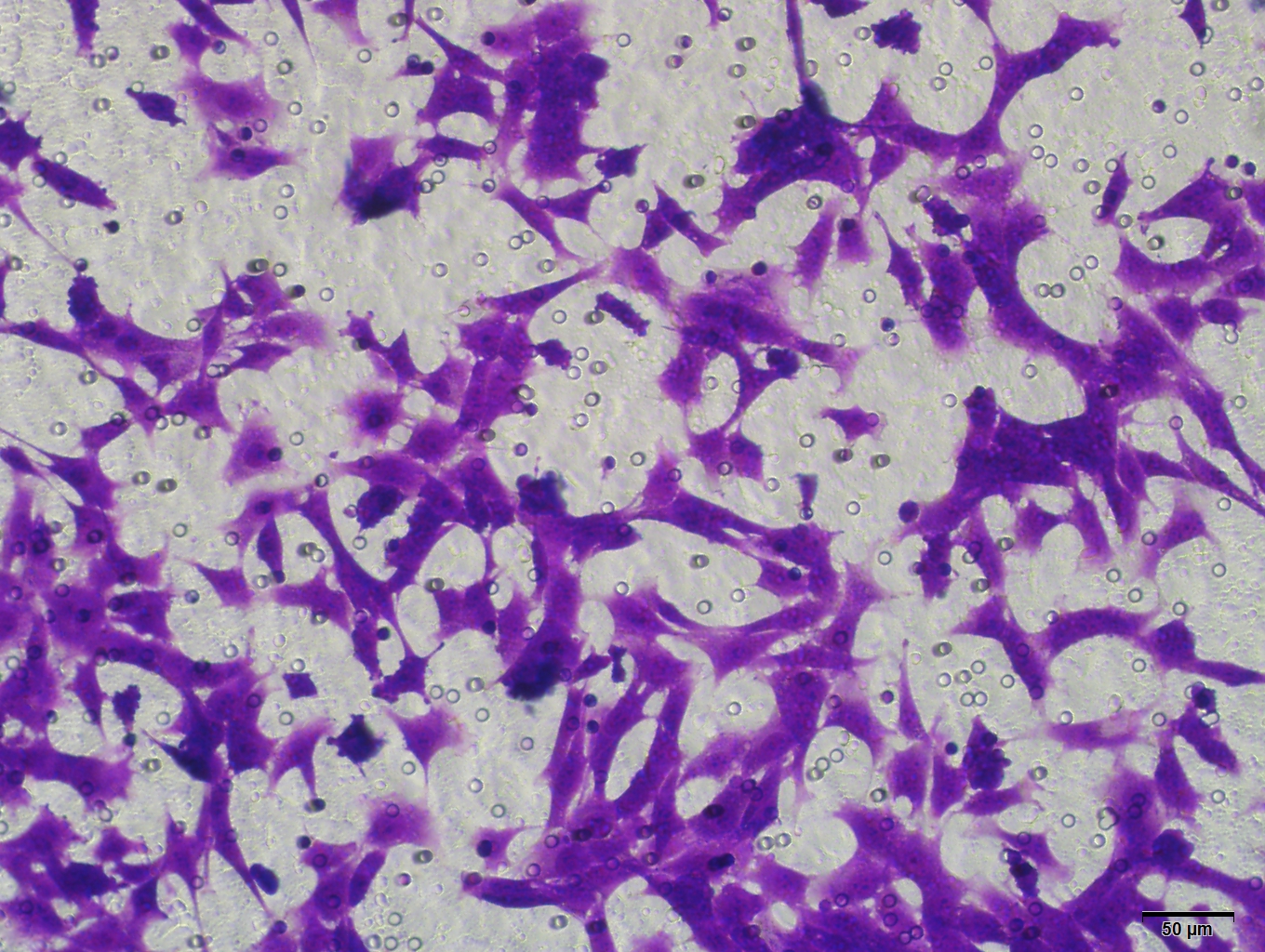

Supplement: Supplemental Information 3 — Figure data and images of WB, wound heal assay, and transwell assay [file peerj-08-9100-s003.zip › Raw data/Tanswell/Fig.7C-miRNA-211-3p.jpg]

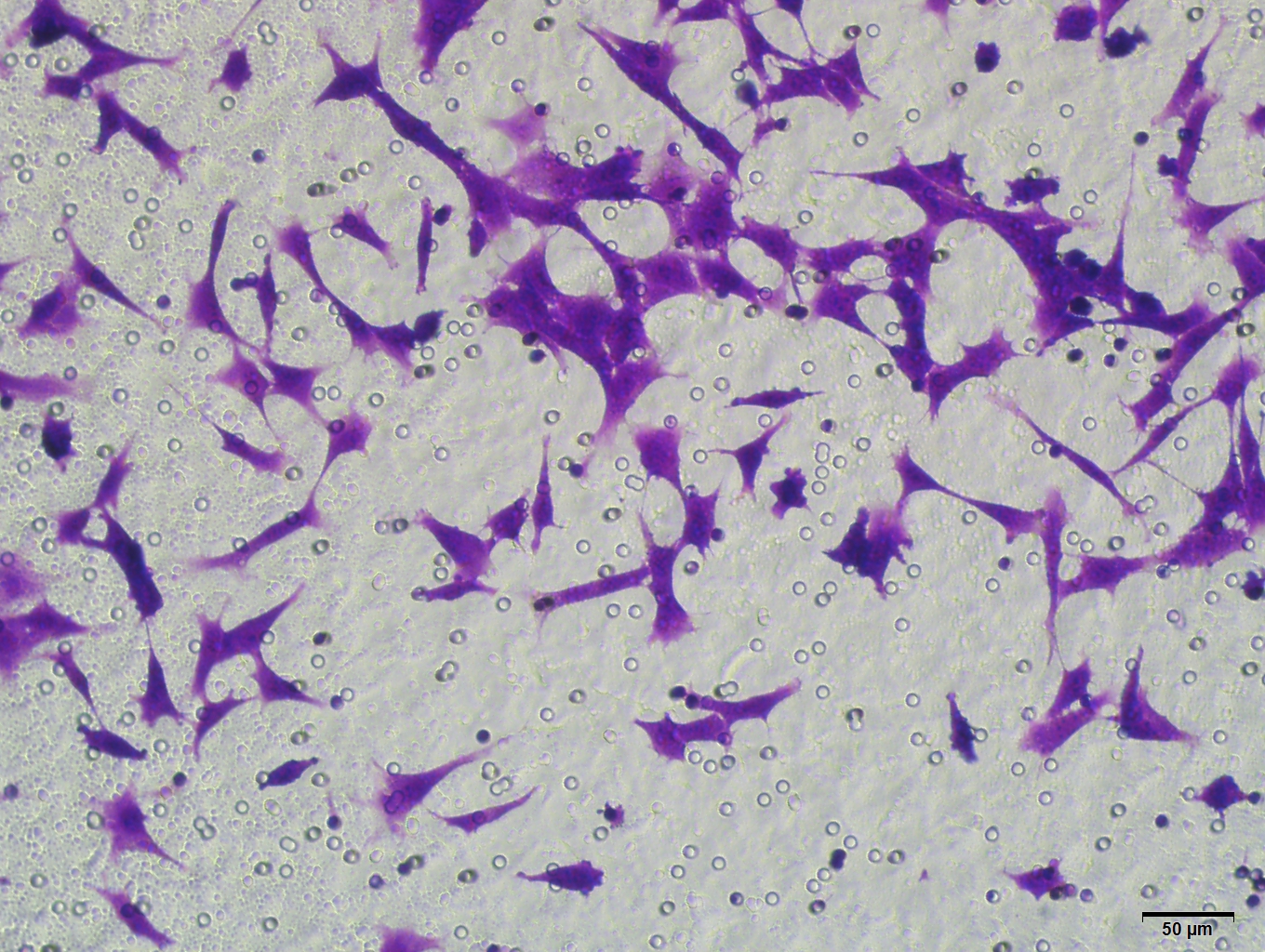

Supplement: Supplemental Information 3 — Figure data and images of WB, wound heal assay, and transwell assay [file peerj-08-9100-s003.zip › Raw data/Tanswell/Fig.7C-miRNA-375.jpg]

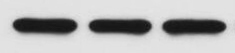

Supplement: Supplemental Information 3 — Figure data and images of WB, wound heal assay, and transwell assay [file peerj-08-9100-s003.zip › Raw data/WB/Fig.5D_GAPDH.jpg]

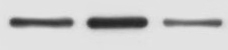

Supplement: Supplemental Information 3 — Figure data and images of WB, wound heal assay, and transwell assay [file peerj-08-9100-s003.zip › Raw data/WB/Fig.5D_TP531.jpg]

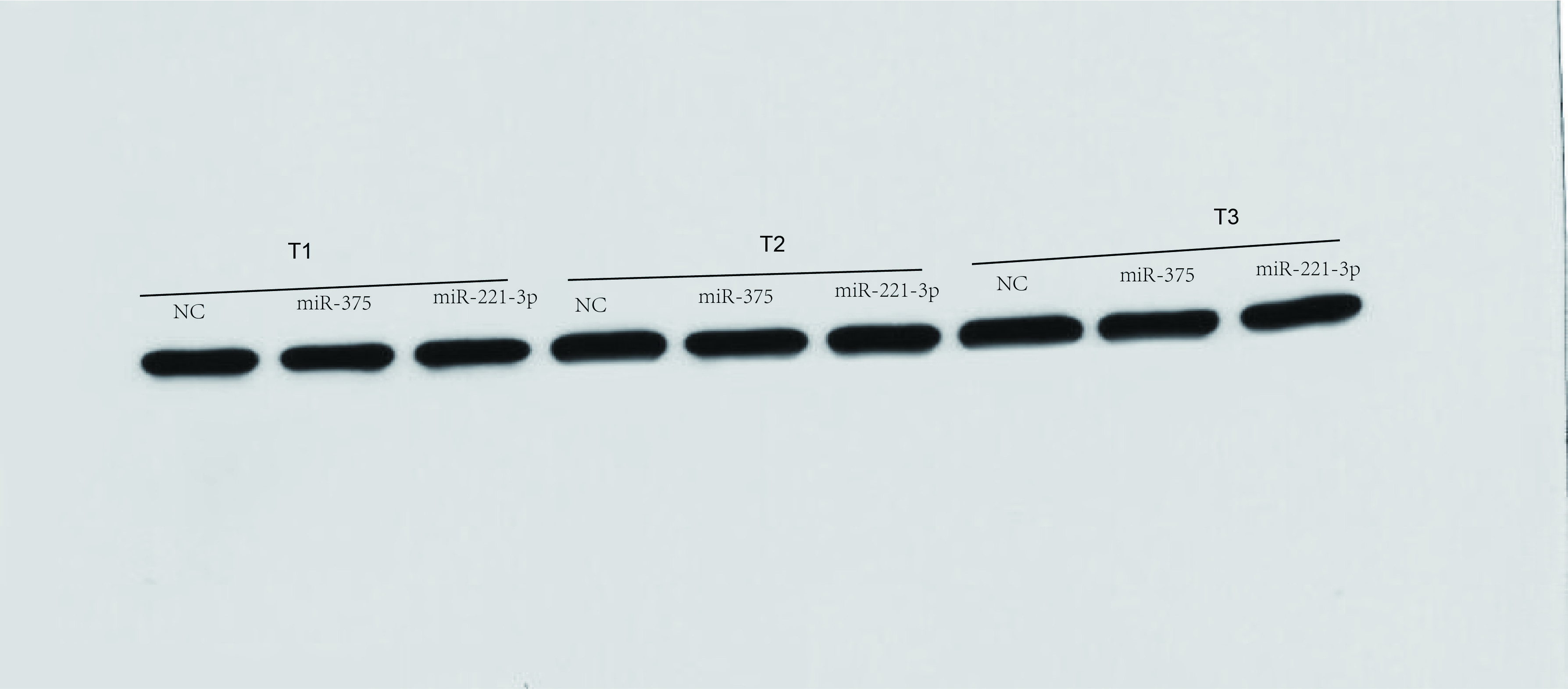

Supplement: Supplemental Information 3 — Figure data and images of WB, wound heal assay, and transwell assay [file peerj-08-9100-s003.zip › Raw data/WB/Tagged_GAPDH.jpg]

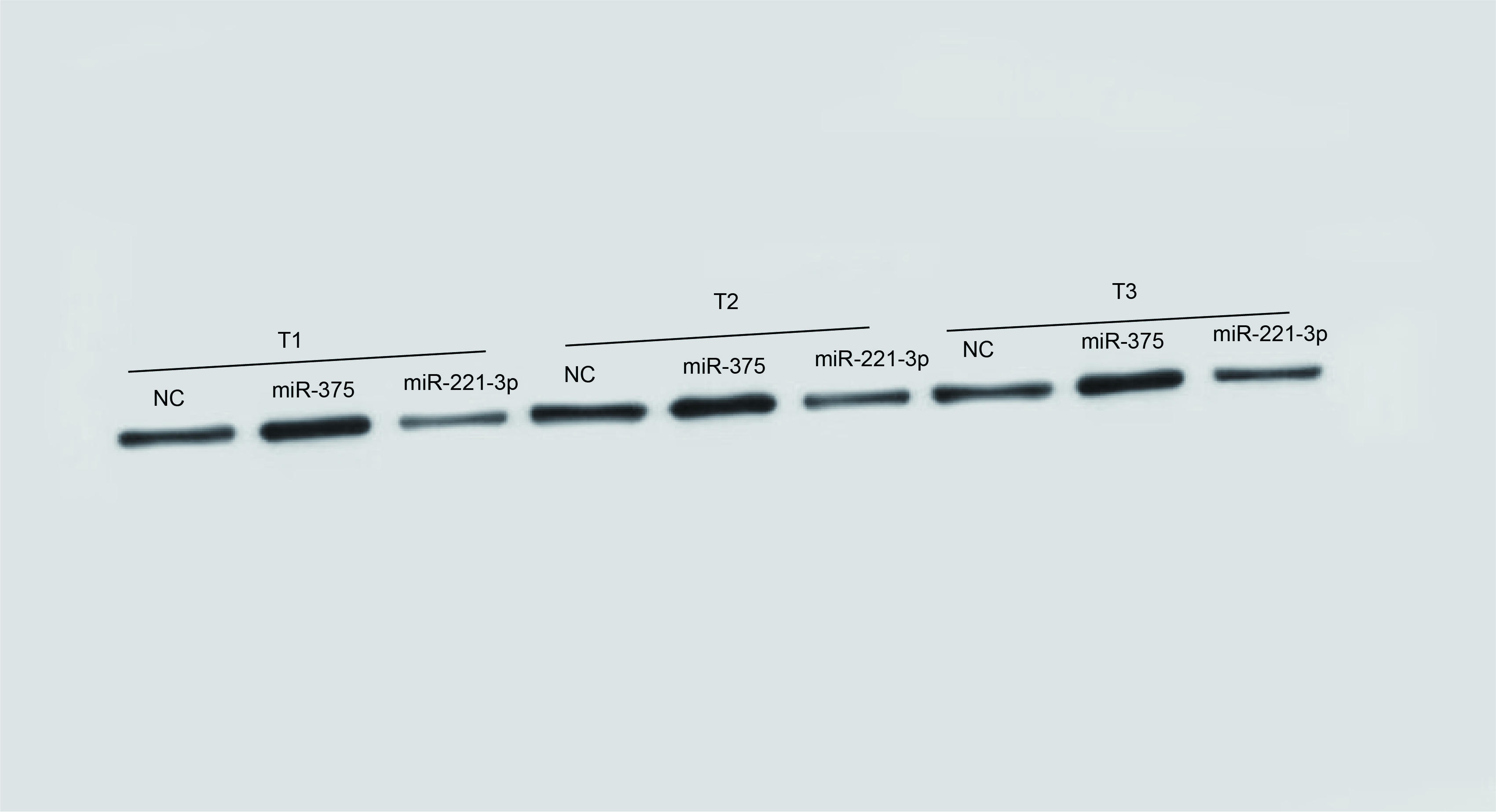

Supplement: Supplemental Information 3 — Figure data and images of WB, wound heal assay, and transwell assay [file peerj-08-9100-s003.zip › Raw data/WB/Tagged_TP53.jpg]

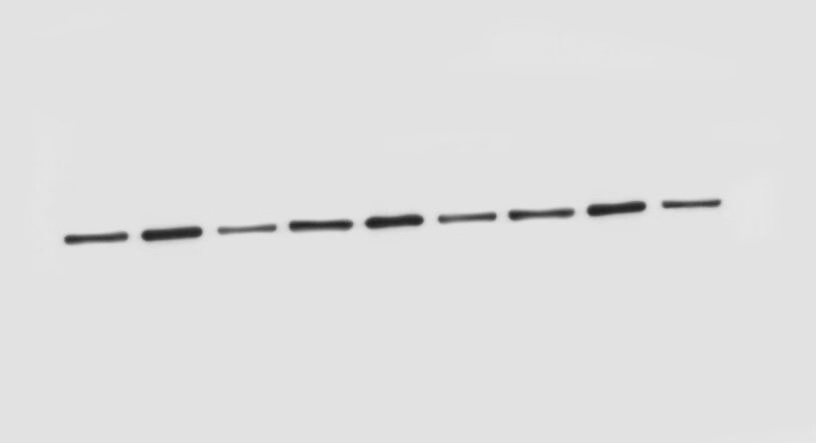

Supplement: Supplemental Information 3 — Figure data and images of WB, wound heal assay, and transwell assay [file peerj-08-9100-s003.zip › Raw data/WB/raw-TP53.jpg]

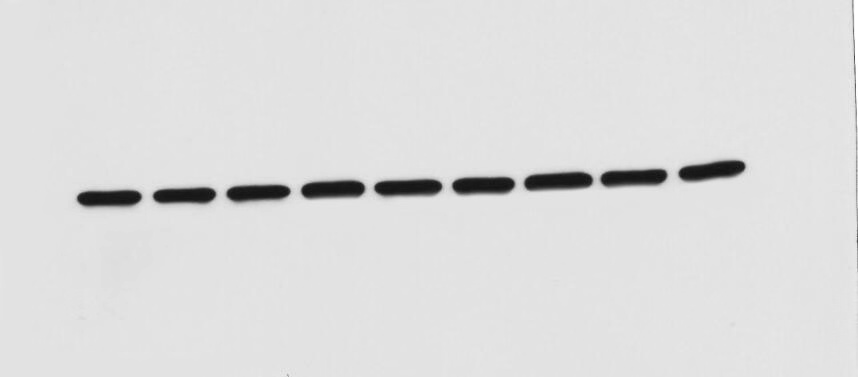

Supplement: Supplemental Information 3 — Figure data and images of WB, wound heal assay, and transwell assay [file peerj-08-9100-s003.zip › Raw data/WB/raw_GAPDH.jpg]

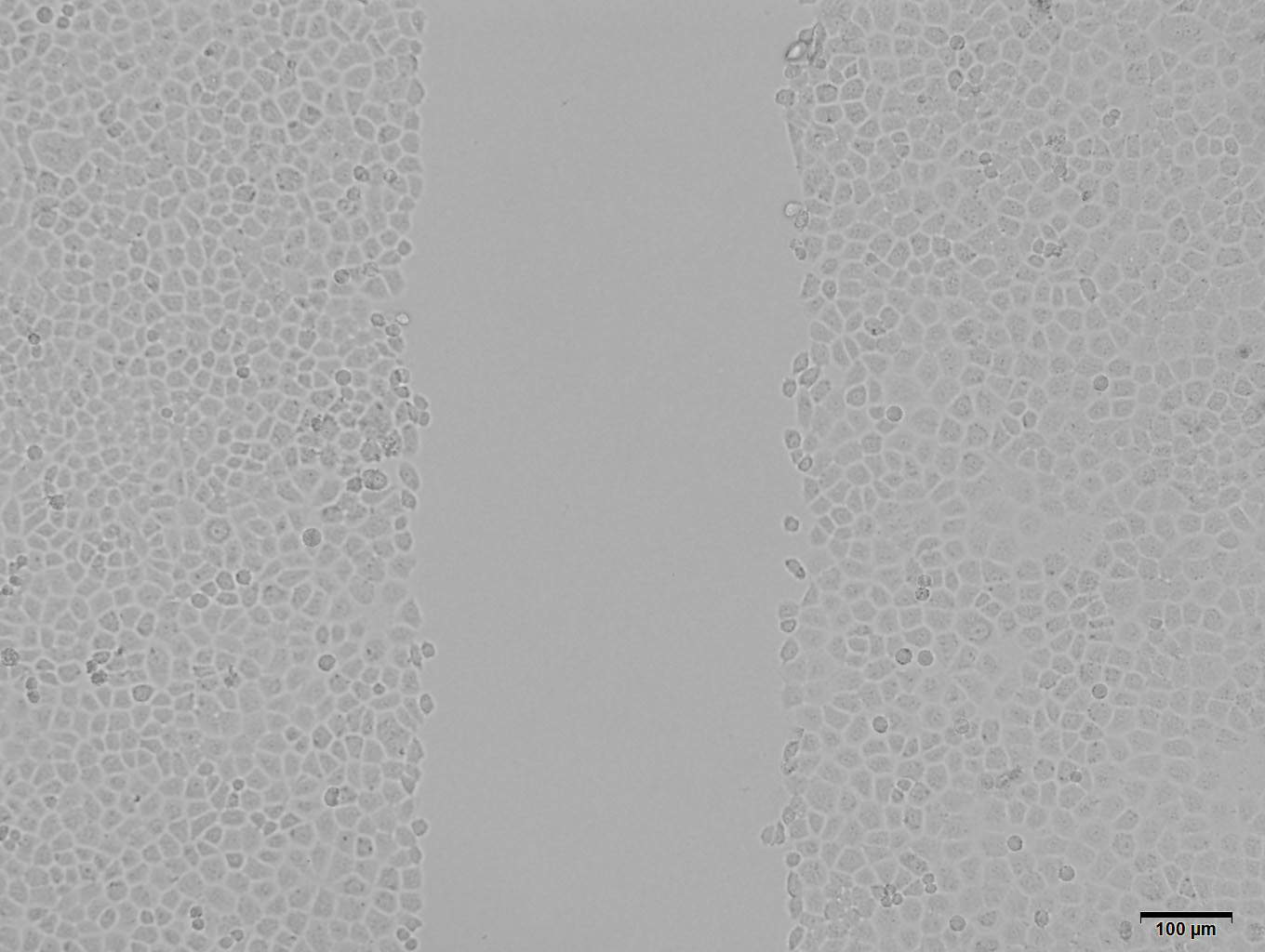

Supplement: Supplemental Information 3 — Figure data and images of WB, wound heal assay, and transwell assay [file peerj-08-9100-s003.zip › Raw data/wound healing/Fig.7A 0h.jpg]

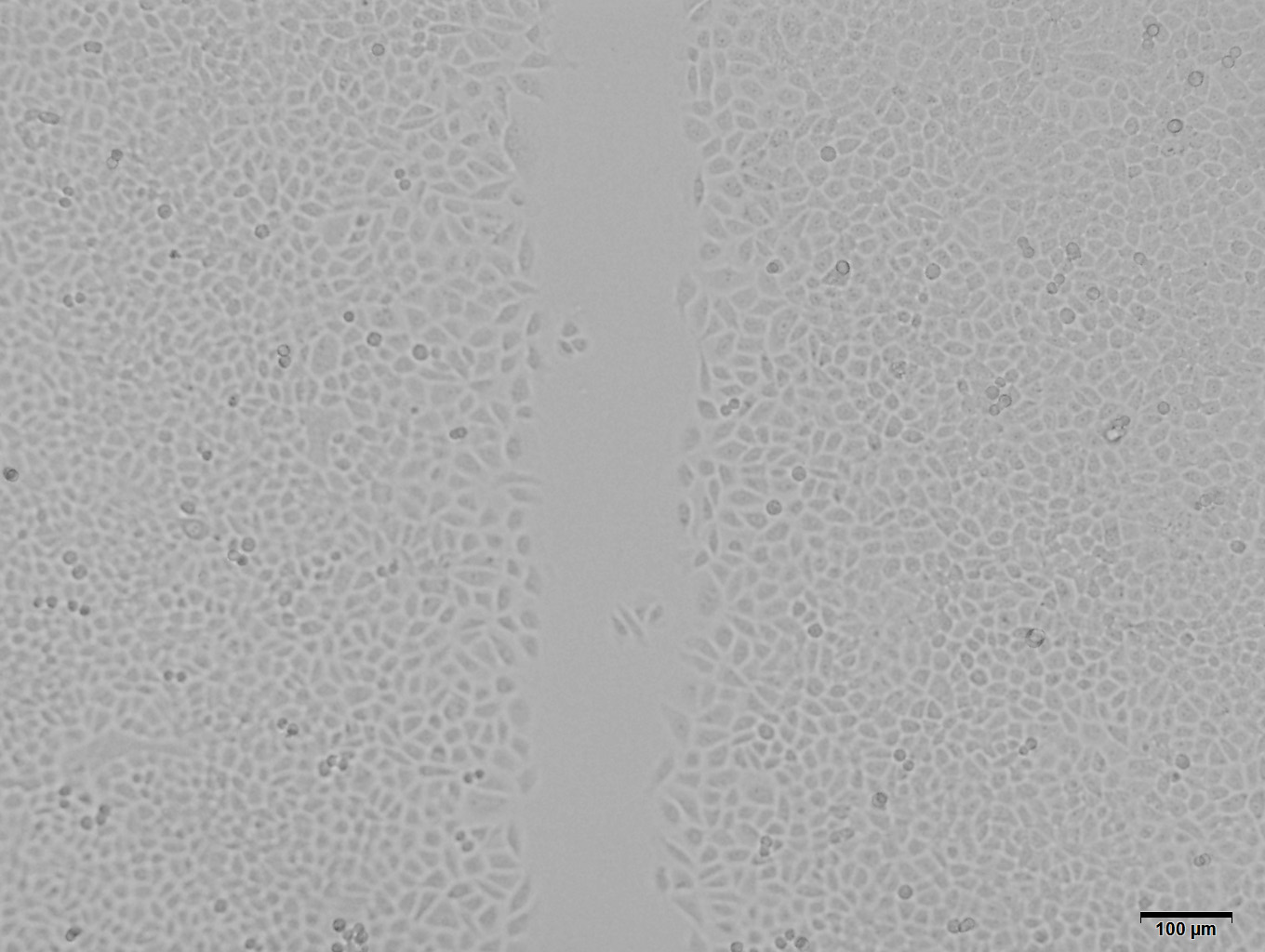

Supplement: Supplemental Information 3 — Figure data and images of WB, wound heal assay, and transwell assay [file peerj-08-9100-s003.zip › Raw data/wound healing/Fig.7A 24h.jpg]

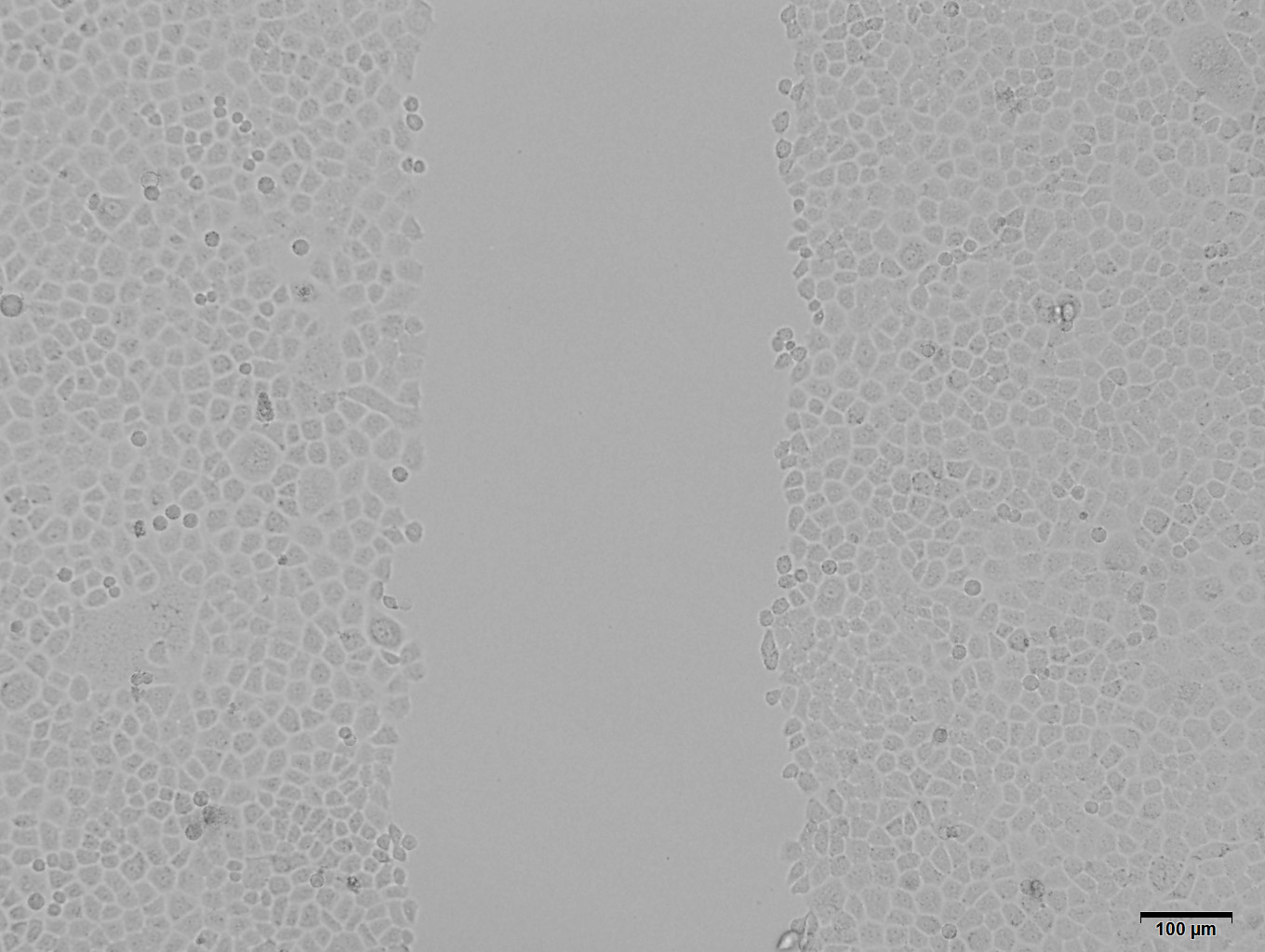

Supplement: Supplemental Information 3 — Figure data and images of WB, wound heal assay, and transwell assay [file peerj-08-9100-s003.zip › Raw data/wound healing/Fig.7A miR-211-3p 0h.jpg]

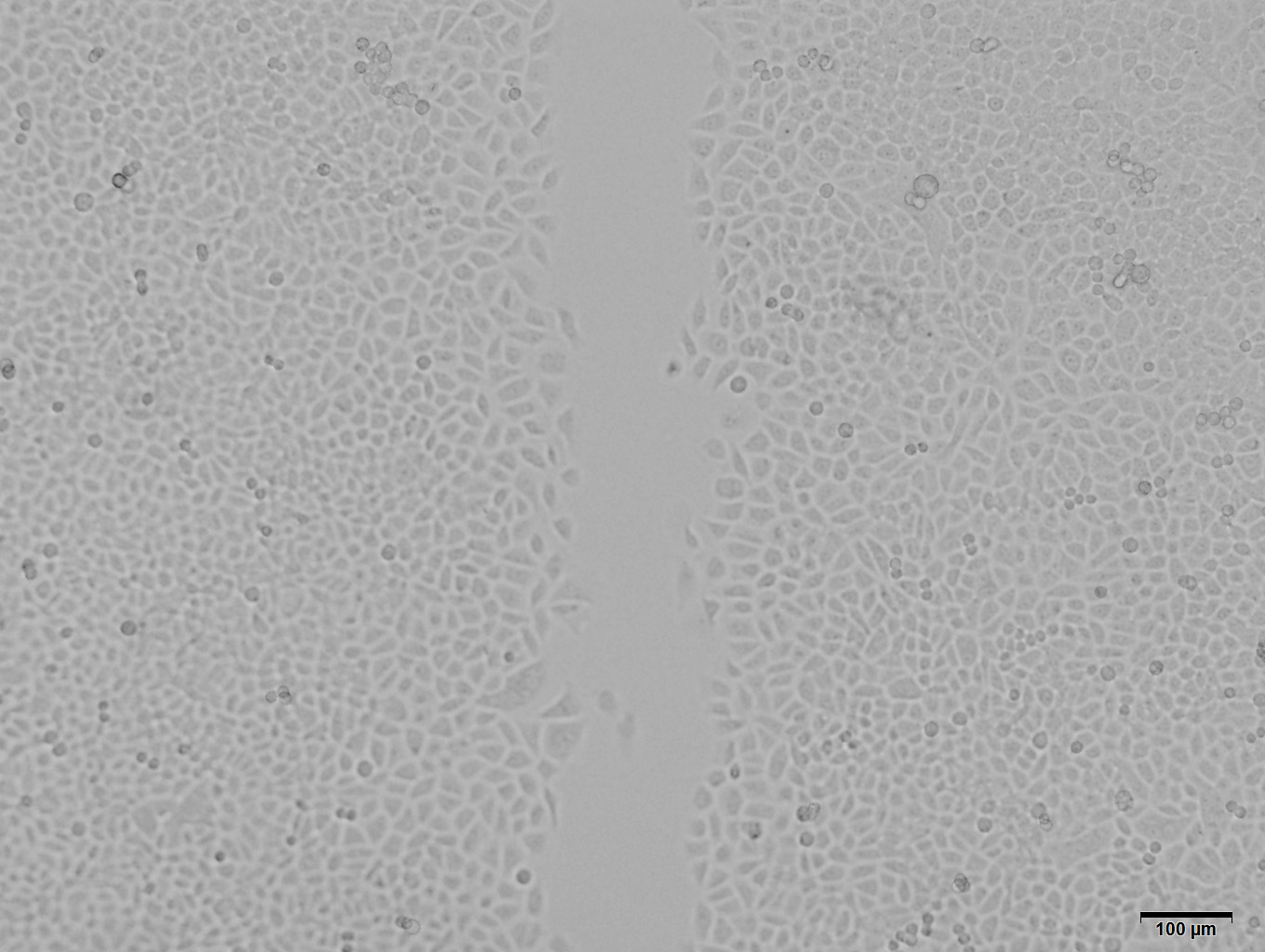

Supplement: Supplemental Information 3 — Figure data and images of WB, wound heal assay, and transwell assay [file peerj-08-9100-s003.zip › Raw data/wound healing/Fig.7A miR-211-3p 24h.jpg]

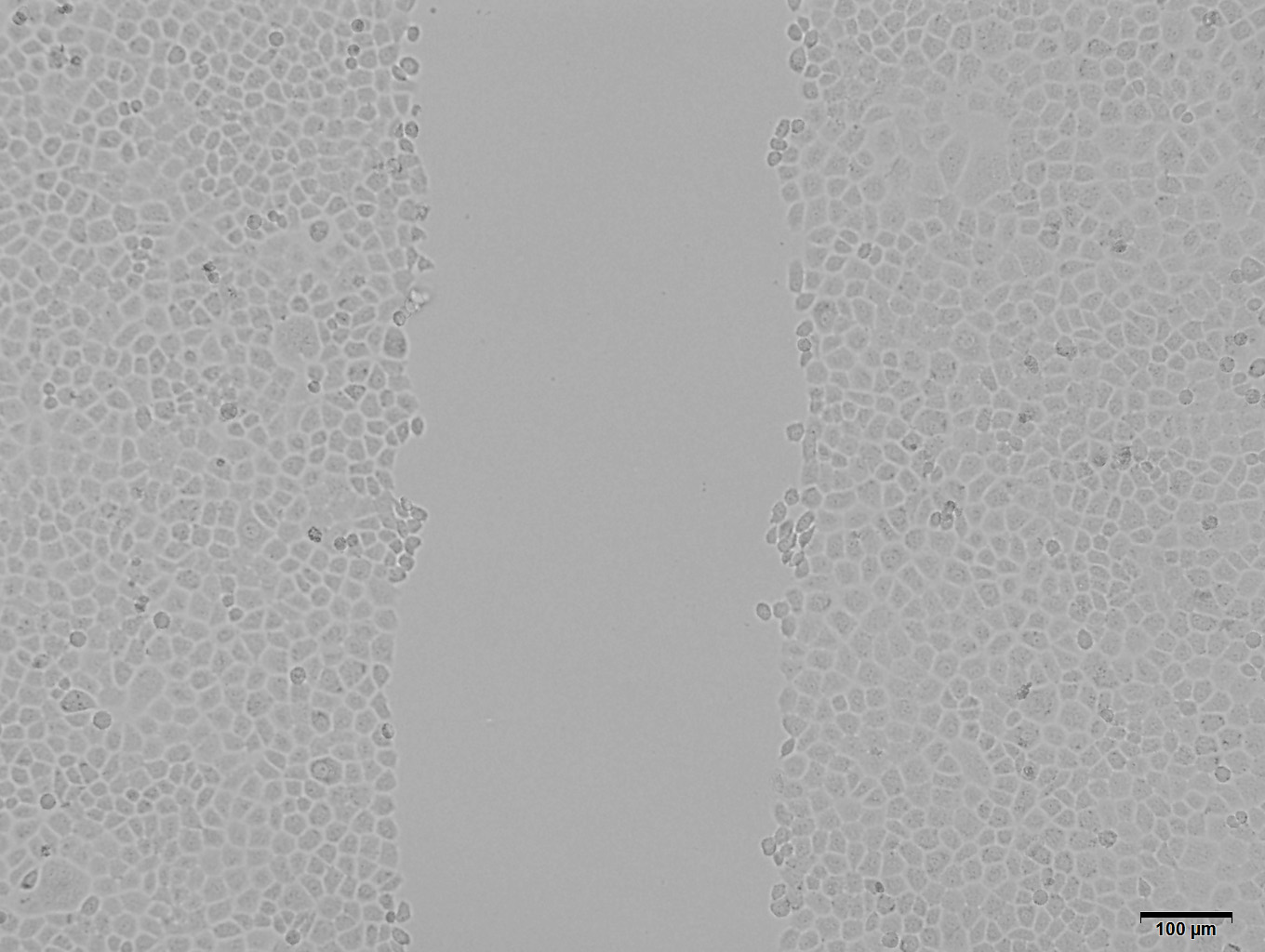

Supplement: Supplemental Information 3 — Figure data and images of WB, wound heal assay, and transwell assay [file peerj-08-9100-s003.zip › Raw data/wound healing/Fig.7A miR-375 0h.jpg]

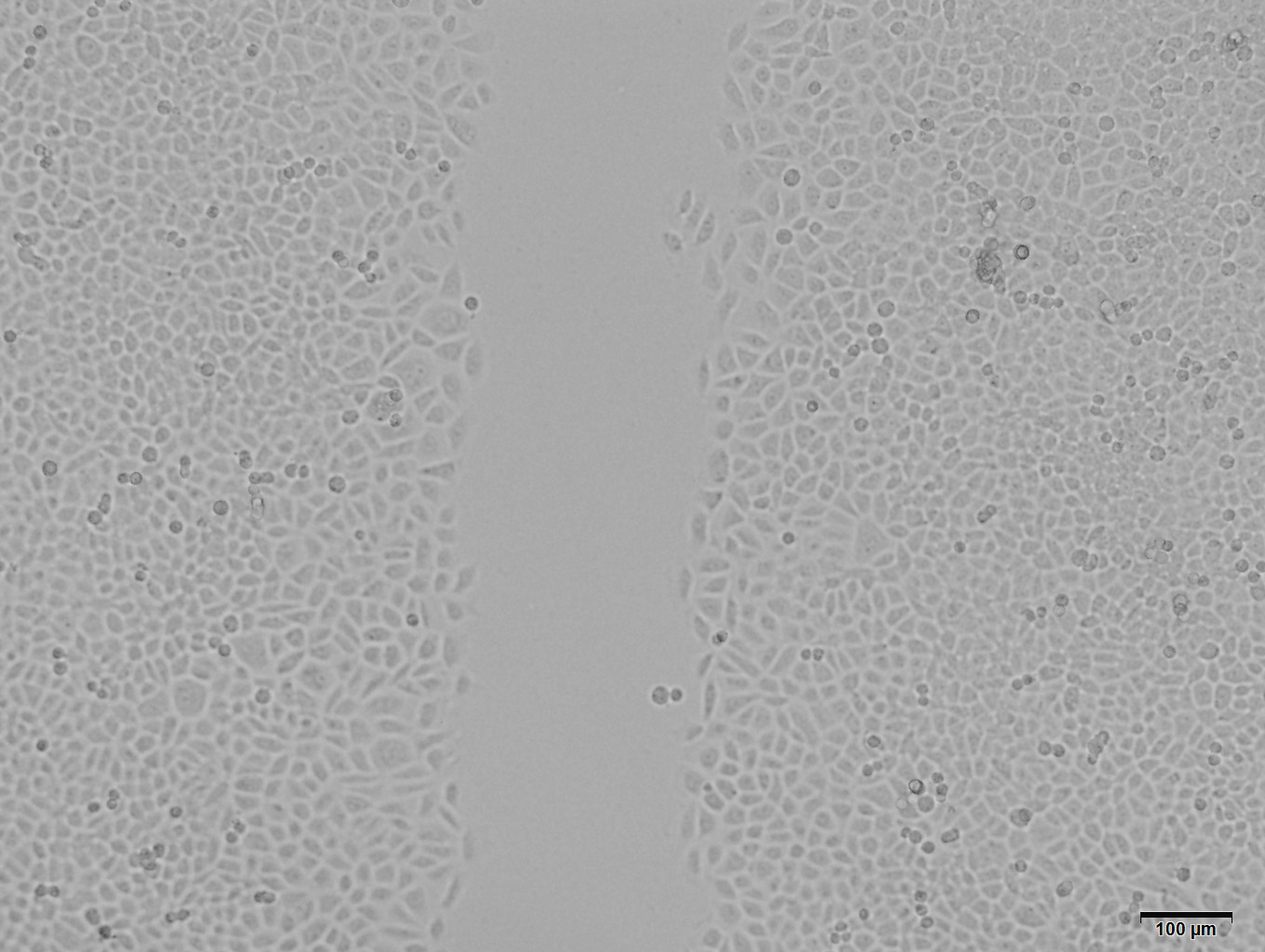

Supplement: Supplemental Information 3 — Figure data and images of WB, wound heal assay, and transwell assay [file peerj-08-9100-s003.zip › Raw data/wound healing/Fig.7A mmiR-375 24h.jpg]
