# Supplementary material for: Aberrant expression of two miRNAs promotes proliferation, hepatitis B virus amplification, migration and invasion of hepatocellular carcinoma cells: evidence from bioinformatic analysis and experimental validation
Source: PeerJ. 2020 Apr 29;8:e9100. doi: 10.7717/peerj.9100 (PMC7195830; doi:10.7717/peerj.9100)
Supplement: Supplemental Information 3 — Figure data and images of WB, wound heal assay, and transwell assay [file peerj-08-9100-s003.zip › Raw data/Fig data.docx]

**Raw data just for review**

| **Fig.5 A** | | | | |
| --- | --- | --- | --- | --- |
| Testing | HL7702 | HepG2 | HepG2.2.15 | MHCC-LM3 |
| T1 | 1.02 | 1.89 | 3.55 | 5.52 |
| T2 | 0.93 | 1.75 | 3.35 | 5.2 |
| T3 | 1.02 | 1.7 | 3.45 | 5.62 |

| **Fig.5 B** | | | | |
| --- | --- | --- | --- | --- |
| Testing | HL7702 | HepG2 | HepG2.2.15 | MHCC-LM3 |
| T1 | 1.02 | 0.8 | 0.52 | 0.22 |
| T2 | 1.05 | 0.88 | 0.46 | 0.24 |
| T3 | 0.92 | 0.79 | 0.43 | 0.28 |

| **Fig.5 C** | | |
| --- | --- | --- |
| Testing | paratumor | tumor |
| T1 | 1.273304 | 1.193904 |
| T2 | 0.637878 | 1.032698 |
| T3 | 0.515029 | 1.528394 |
| T4 | 0.673321 | 1.220596 |
| T5 | 1.337301 | 0.824195 |
| T6 | 1.352253 | 0.813626 |
| T7 | 1.335942 | 1.737615 |
| T8 | 0.656357 | 0.912031 |
| T9 | 1.159242 | 0.876442 |
| T10 | 1.38036 | 1.465146 |
| T11 | 0.528635 | 1.590442 |
| T12 | 0.57528 | 1.762039 |
| T13 | 1.431616 | 1.400646 |
| T14 | 1.199912 | 1.364553 |
| T15 | 0.98506 | 0.995817 |
| T16 | 1.276196 | 0.960178 |
| T17 | 1.325742 | 1.290008 |
| T18 | 1.4263 | 1.429839 |
| T19 | 0.589543 | 1.748162 |
| T20 | 0.679216 | 1.237741 |

| **Fig.5 D** | | |
| --- | --- | --- |
| Testing | paratumor | tumor |
| T1 | 0.895475 | 0.258089 |
| T2 | 0.79081 | 0.281283 |
| T3 | 0.996393 | 0.406359 |
| T4 | 0.829042 | 0.292174 |
| T5 | 0.788486 | 0.345422 |
| T6 | 1.03948 | 0.410409 |
| T7 | 1.194675 | 0.385717 |
| T8 | 0.760395 | 0.295015 |
| T9 | 0.814564 | 0.369929 |
| T10 | 0.952976 | 0.438382 |
| T11 | 0.992483 | 0.260951 |
| T12 | 1.018523 | 0.255628 |
| T13 | 1.011634 | 0.36214 |
| T14 | 1.071694 | 0.26644 |
| T15 | 1.230277 | 0.328093 |
| T16 | 1.209365 | 0.258972 |
| T17 | 0.993577 | 0.415143 |
| T18 | 0.954571 | 0.413605 |
| T19 | 1.054619 | 0.435374 |
| T20 | 1.051401 | 0.441861 |

**Fig.5 E from OncomiR dataset http://www.oncomir.org/**


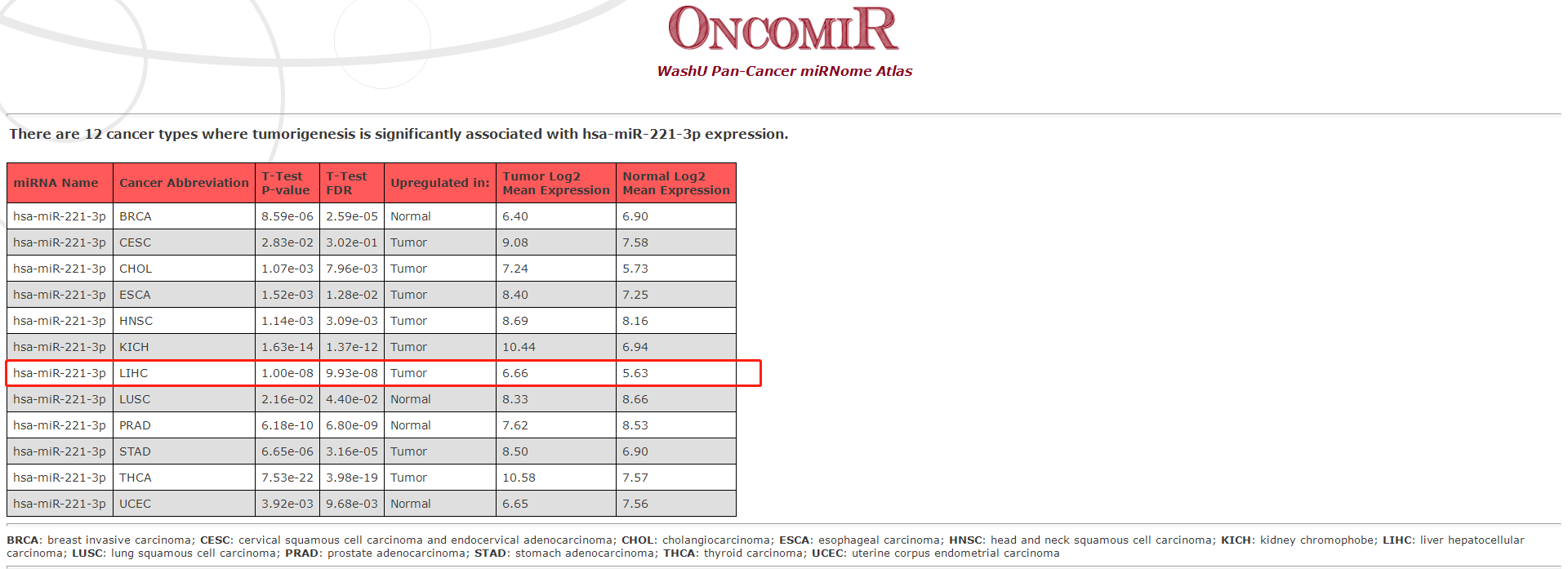


**Fig.5 F from OncomiR dataset http://www.oncomir.org/**


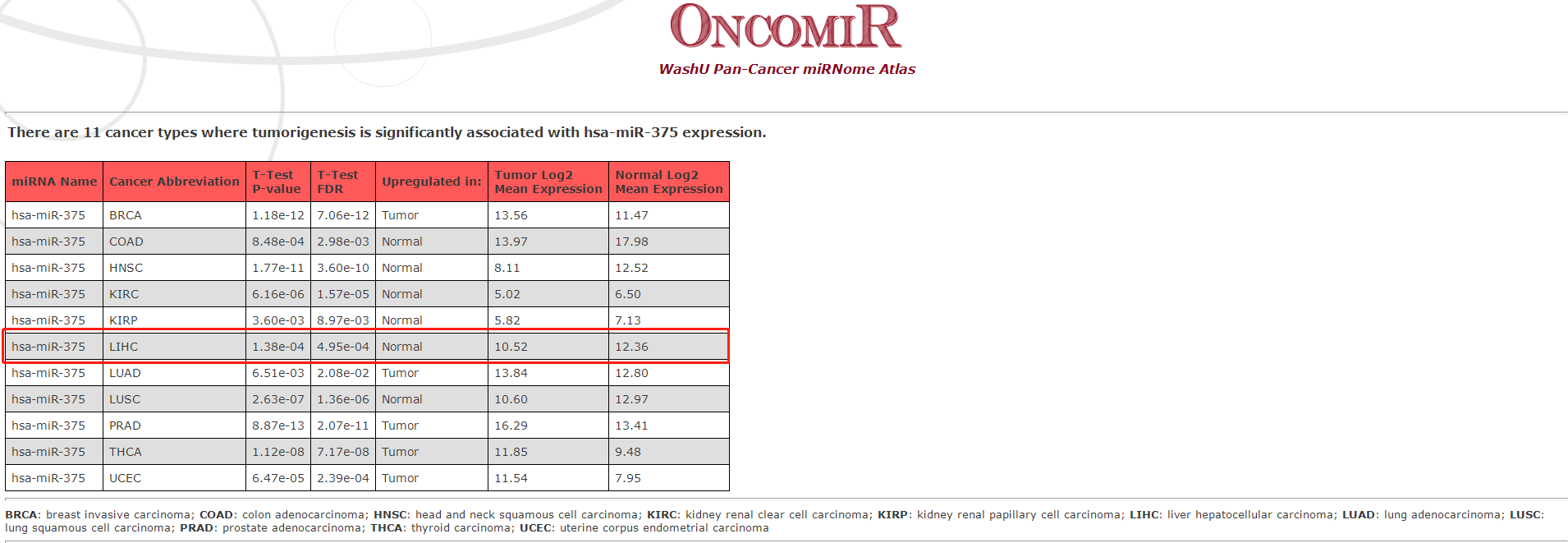


**Fig.5 G from OncomiR dataset http://www.oncomir.org/**


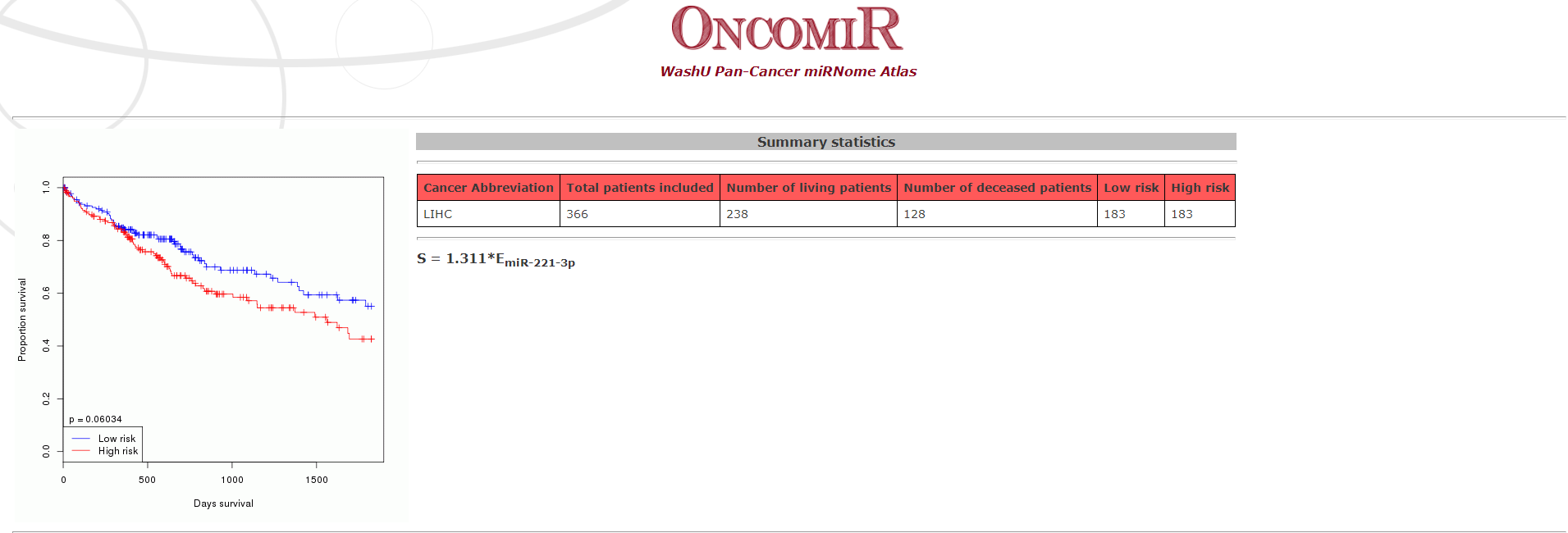


**Fig.5 H from OncomiR dataset http://www.oncomir.org/**


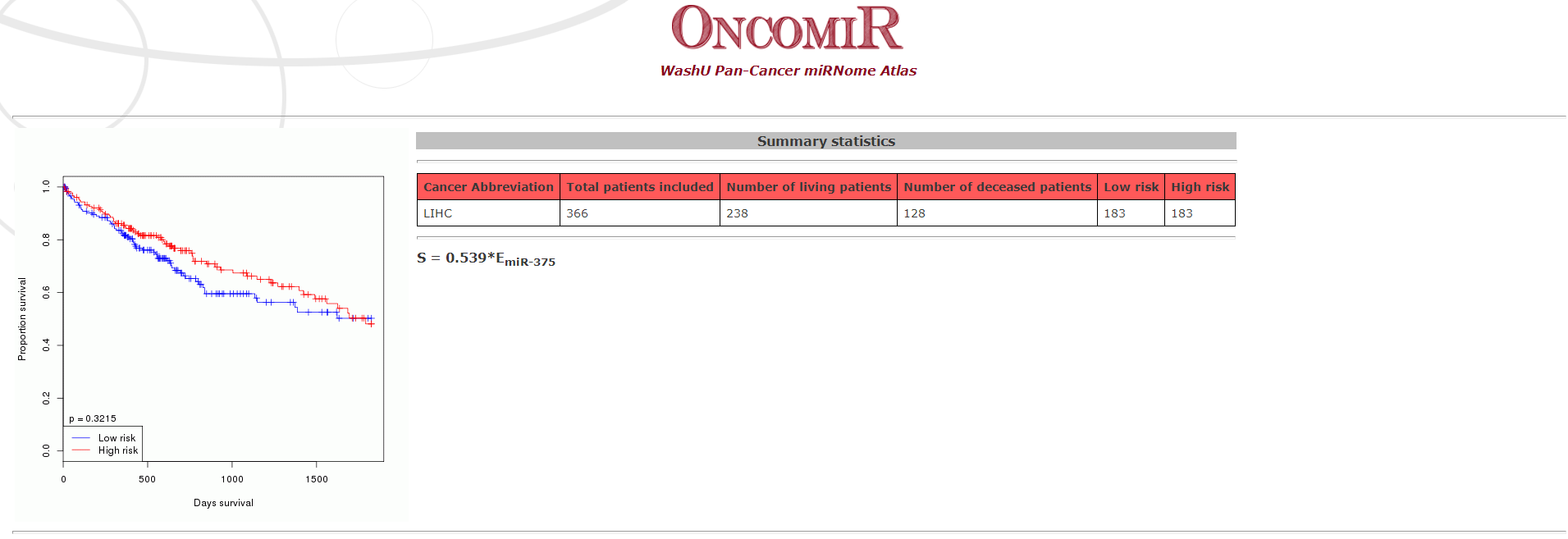


| **Fig.6 A** | | | | |
| --- | --- | --- | --- | --- |
|  | miR-211-3p | | miR-375 | |
| Testing | 50nM | 100nM | 50nM | 100nM |
| T1 | 620 | 683 | 820 | 1083 |
| T2 | 602 | 650 | 802 | 1050 |
| T3 | 595 | 662 | 755 | 1032 |

| **Fig.6 B** | | | |
| --- | --- | --- | --- |
| Testing | NC | miR-7-5p | miR-375 |
| T1 | 0.564 | 0.965 | 0.321 |
| T2 | 0.595 | 0.92 | 0.305 |
| T3 | 0.486 | 0.87 | 0.248 |

| **Fig.6 C** | | | |
| --- | --- | --- | --- |
| Testing | NC | miR--221-3p | miR-375 |
| T1 | 5.42 | 5.92 | 4.02 |
| T2 | 5.56 | 6.02 | 4.26 |
| T3 | 5.3 | 5.8 | 4.33 |

**Fig.7 B**

| NC | miR-7-5p | miR-375 |
| --- | --- | --- |
| 48.5 | 78.6 | 35.6 |
| 45.8 | 82.3 | 33.4 |
| 52.3 | 75.5 | 30.2 |

**Fig.7 D**

| NC | miR-7-5p | miR-375 |
| --- | --- | --- |
| 152 | 247 | 78 |
| 164 | 236 | 82 |
| 143 | 260 | 65 |
